# Lack of p53 Affects the Expression of Several Brain Mitochondrial Proteins: Insights from Proteomics into Important Pathways Regulated by p53

**DOI:** 10.1371/journal.pone.0049846

**Published:** 2012-11-27

**Authors:** Ada Fiorini, Rukhsana Sultana, Eugenio Barone, Giovanna Cenini, Marzia Perluigi, Cesare Mancuso, Jian Cai, Jon B. Klein, Daret St. Clair, D. Allan Butterfield

**Affiliations:** 1 Department of Biochemical Sciences, Sapienza University of Rome, Rome, Italy; 2 Department of Chemistry, Center of Membrane Sciences, Sanders Brown Center on Aging, University of Kentucky, Lexington, Kentucky, United States of America; 3 Institute of Pharmacology, Catholic University School of Medicine, Rome, Italy; 4 Division of Nephrology, Department of Medicine and Proteomics Center, University of Louisville, Louisville, Kentucky, United States of America; 5 Graduate Center for Toxicology, University of Kentucky, Lexington, Kentucky, United States of America; Oregon Health & Science University, United States of America

## Abstract

The tumor suppressor protein p53 has been described “as the guardian of the genome” for its crucial role in regulating the transcription of numerous genes responsible for cells cycle arrest, senescence, or apoptosis in response to various stress signals. Although p53 promotes longevity by decreasing the risk of cancer through activation of apoptosis or cellular senescence, several findings suggest that an increase of its activity may have deleterious effects leading to selected aspects of the aging phenotype and neurodegenerative diseases. There is the link between p53 and oxidative stress, the latter a crucial factor that contributes to neurodegenerative processes like Alzheimer disease (AD). In the present study, using a proteomics approach, we analyzed the impact of lack of p53 on the expression of several brain mitochondrial proteins involved in different pathways, and how lack of p53 may present a target to restore neuronal impairments. Our investigation on isolated brain mitochondria from p53^(−/−)^ mice also provides a better understanding of the p53-mitochondria relationship and its involvement in the development of many diseases.

## Introduction

The p53 tumor suppressor protein plays a central role to preserve genomic integrity [1] with effect on cell fate [2]. p53 is involved in many cellular pathways, and when this protein becomes activated in response to stress signals [3] it can promote a transient cell cycle arrest, cell death (apoptosis) or permanent cell cycle arrest (senescence) [4]. p53 often is lost or mutated in cancers [5]. Both apoptosis and cellular senescence prevent the propagation of damaged DNA [6] with consequent reduction of the risk of cancer. However, both of these processes favor tissue atrophy and aging phenotype [7]. Therefore, p53 can exert both beneficial and deleterious effects depending on a delicate balance between tumor suppressor and longevity.

The interaction among p53 and oxidative stress is intriguing, since this latter is well known to be associated with several age-related diseases [8], [9]. Under normal conditions, p53 protein levels are low and regulated by IKK but prominently by Mdm2, an ubiquitin ligase responsible for p53 degradation. Cellular stress reduces the interaction between p53 and Mdm2 leading to accumulation of the former [10], and several reactive oxygen (ROS) and nitrogen species (RNS) also modify p53 and its activity [11]. Moreover, the activation of p53 leads to the generation of ROS as well [12], [13]. Thus, there is an intricate link between p53 and ROS, even though specific mechanisms of their interplay are still unclear. Several results show that cellular redox status is under control of p53, and p53 may exert opposite effects in ROS regulation depending on its levels [11]. Physiological levels of p53 maintain ROS at basal levels through transactivation of antioxidant genes such as SESN1 (mammalian sestrin homologue), SESN2, and glutathione peroxidase-1 (GPx1) [14]. In addition, constitutive levels of p53 link energy metabolism to ROS formation by regulating the expression of essential metabolic enzymes that are able to balance energy metabolism among mitochondrial respiration, glycolysis, and the pentose phosphate shunt [11], and mitochondrial respiration is a major source of ROS [15], [16].

High levels of p53 increase intracellular ROS by transactivation of genes encoding pro-oxidant proteins such as NQO1 (quinone oxidoreductase) [11] and proline oxidase (POX) [11], and for pro-apoptotic proteins, which include BAX and PUMA [11]. Further, the repression of antioxidant enzymes such as MnSOD by p53, is another means to increase intracellular ROS [11], [17].

**Figure 1:**
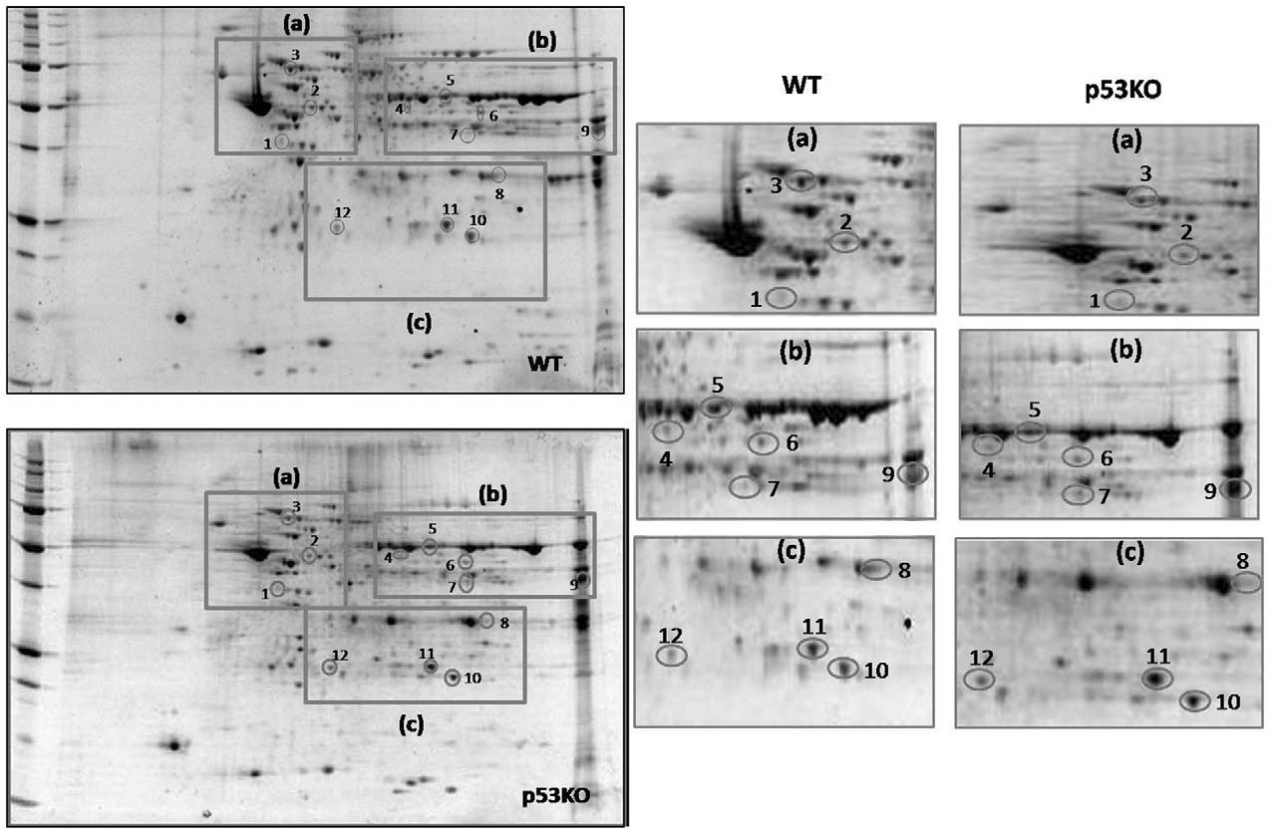
Proteomic analysis of differential protein expression (WT vs. p53KO). Proteomic profile of representative 2D-gels with proteins differently expressed between mitochondrial fraction isolated from the brain of WT mice and p53^(−/−)^ (left); expanded images of protein spots that have significantly different levels (p<0.05) between WT and p53^(−/−)^ (right).

Changes in mitochondrial ROS production may influence the p53 pathway [18], [19]. Also p53 can regulate ROS production in mitochondria [20]. This suggests that there is an interaction between mitochondria and p53 essential to allow normal cellular functions and its interruption may have severe consequences [21]. Consequently, understanding better the mechanisms underlying this interaction may be helpful to further comprehend the development and the progression of many diseases [21].

**Table 1:** Proteins Expressed Differently in Mitochondrial Fraction Isolated from the Brain of WT and p53^(−/−)^ mice.

Spot	Protein Identified	Accession #	Coverage	Number of identified peptides^a^	Score	MW (kDa)	pI	P value^b^	Fold^c^
1	Guanine nucleotide-binding protein G (o) subunit alpha	P18872	12.15	3	24.11	40.1	5.53	0.0019	212 ↑ p53KO
2	ATP synthase subunit beta, mitochondrial	P56480	4.54	2	18.16	56.3	5.34	0.0035	125 ↑ p53KO
3	Heat shock cognate 71 kDa protein	P63017	37.31	20	196.60	70.8	5.52	0.002	212 ↑ p53KO
4	Aldehyde dehydrogenase family 5, subfamily A1	B2RS41	14.72	6	36.70	55.9	8.25	0.0009	131 ↑ p53KO
5	Glutamate dehydrogenase 1, mitochondrial	P26443	26.34	13	78.69	61.3	8.00	0.0076	131 ↑ p53KO
6	Isoform mithocondrial of Fumarate hydratase	P97807-2	25.57	8	62.73	50.0	7.94	0.0019	325 ↑ p53KO
7	Acetyl-CoA acetyltransferase	Q8QZT1	26.89	8	50.64	44.8	8.51	0.00079	166 ↑ 53KO
8	Isoform Mt-VDAC1 of Voltage- dependent anion-selective channel protein 1	Q60932-2	38.16	7	74.55	30.7	8.54	0.0027	201 ↑ p53KO
9	Aspartate aminotransferase	P05202	43.72	17	174.33	47.4	9.00	0.0037	210 ↑ p53KO
10	Mn Superoxide dismutase	P09671	13.96	4	43.39	24.6	8.62	0.0026	133 ↑ 53KO
11	Cytochrome b-c1 complex Rieske subunit	Q9CR68	26.28	7	70.31	29.3	8.70	0.0030	252 ↑ 53KO
12	Thioredoxin-dependent peroxide reductase	P20108	28.40	7	41.17	28.1	7.58	0.0015	253 ↑ 53KO

a The number of peptide sequences identified by nanospray ESI-MS/MS of tryptic peptides.

b The fold-change in spot density from p53^(−/−)^ mice compared to wt. The arrow indicates the direction of change.

c The p-value associated with fold-change calculated using a Student's t-test.

The aim of this study was to analyze the impact that the lack of p53 had on basal protein expression levels in mitochondria isolated from mice brain, to gain insight into the special link between p53 and oxidative stress, and its impact on neurodegenerative disorders, such as Alzheimer disease. A proteomics approach was used.

**Table 2:** Functionalities of Identified Proteins Differently Expressed.

Functions	Proteins involved
Energy or mitochondrial alterations	ATP synthase subunit beta, mitochondrial Aldehyde dehydrogenase family 5, subfamily A1 Glutamate dehydrogenase 1, mitochondrial Isoform mitochondrial of Fumarate hydratase Acetyl-CoA acetyltransferase VDAC1 of Voltage-dependent anion-selective channel protein 1 Aspartate aminotransferase Mn Superoxide dismutase Cytochrome b-c1 complex Rieske subunit
Signal transduction	Guanine nucleotide-binding protein G (o) subunit alpha
Antioxidant defence/detoxification dysfunction	Mn Superoxide dismutase Thioredoxin-dependent peroxide reductase
Chaperone proteins	Heat shock cognate 71 kDa protein

## Materials and Methods

### Chemicals

All chemicals used in this study were purchased from Bio-Rad (Hercules, CA).

**Figure 2:**
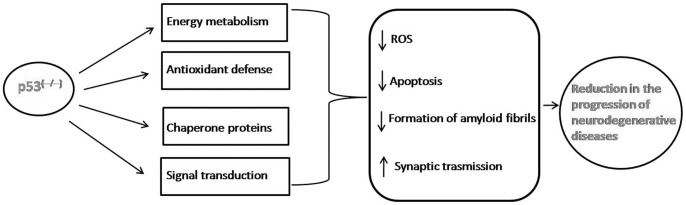
Putative network of pathways regulated by p53KO. A model of how the lack of p53 affects biological pathways that would attenuate progression of neurodegenerative disorders. Our result potentially makes p53 a novel therapeutic target for the delay, treatment, or prevention of these diseases.

### Animals

Heterozygous mice p53^(−/+)^ were maintained in our laboratory to generate p53^(−/−)^ and wt littermates. p53^(−/−)^ are in the C57BL/6 background and were initially produced in the laboratory of Dr. Tyler Jacks at the Center for Cancer Research and Department of Biology, Massachusetts Institute of Tecnology (Cambridge, MA). The targeted disrupted p53 genes do not yield p53 protein, because of 40% of their gene-coding region is eliminated by the induced mutation. Male mice with an age between 10 and 12 weeks old were used in our study. All animal experimental procedures were approved by the Institute Animal Care and Use Committe of the University of Kentucky and followed NIH Guidelines for the Care and Use of Laboratory Animals.

### Sample preparation

Mice were humanely euthanized, and the brain was quickly removed. Mitochondria were promptly isolated from the brain by differential centrifugation methods using Percoll Gradientswith some modifications [22].

### Isoelectric focusing (IEF)

Proteins from mitochondrial homogenates (200 μg) were precipitated by addition of ice-cold 100% trichloroacetic acid (TCA) (15% final concentration) and incubated on ice for 10 min. Samples were centrifuged at 14,000 rpm (23,700× g) for 5 min at 4°C. Pellets were washed three times with 0.5 mL of wash buffer [1∶1 (v/v) ethanol: ethyl acetate] to remove excess salts. After the final wash, pellets were dried at room temperature (RT) for ∼10 min and rehydrated for 2 h at RT in 200 μl of a rehydration buffer [8 M urea, 2 M thiourea, 50 mM DTT, 2.0% (w/v) CHAPS, 0.2% Biolytes, Bromophenol Blue], placed in agitation for 3 hours, and then sonicated for 10 s. Samples (200 μg) were applied to 11 cm pH 3–10 ReadyStrip™ IPG strips and after 2 h, 2 ml of mineral oil was added to prevent sample evaporation. Strips were actively rehydrated at 20°C for 18 h at 50 V, focused at a constant temperature of 20°C beginning at 300 V for 2 h, 500 V for 2 h, 1000 V for 2 h, 8000 V for 8 h, and finishing at 8000 V for 10 h rapidly. IPG strips were stored at −80°C until the second dimension of analysis was carried out.

### Two-dimensional polyacrylamide gel electrophoresis (2D-PAGE)

2D-PAGE was performed to separate proteins on IEF strips based on molecular migration rate. IEF strips were thawed and equilibrated for 10 min in equilibration buffer A [50 mM Tris–HCl, pH 6.8, 6 M urea, 1% (w/v) SDS, 30% v/v glycerol, 0.5% DTT] and then re-equilibrated for 10 min in equilibration buffer B [50 mM Tris–HCl, pH 6.8, 6 M urea, 1% (w/v) SDS, 30% v/v glycerol, 4.5% IA]. Criterion precast linear gradient (8–16%) Tris–HCl polyacrylamide gels were uesd to perform second dimension electrophoresis. Precision Plus Protein™ All Blue Standards and samples were run at a constant voltage of 200 V for 65 min.

### SYPRO Ruby® staining

After 2D-PAGE, gels were incubated in a fixing solution [7% (v/v) acetic acid, 10% (v/v) methanol] for 20 min at RT. Sypro Ruby® Protein Gel Stain (∼50 ml) was added to gels to stain them overnight at RT on a gently rocking platform. Gels then were placed in deionized water at RT until scanning. Gels were scanned into Adobe Photoshop 6.0 with a Molecular Dynamics STORM Phosphoimager (λex/λem: 470/618 nm) and stored in deionized water at 4 °C until further use.

### Image Analysis

#### Differential expression

Spot intensities from *SYPRO* Ruby®-stained 2D-gel images of WT and p53^(−/−)^ samples were quantified by densitometry according to the total spot density using PD Quest analysis software from Bio-Rad (Hercules, CA). Intensities were normalized to total gel densities and/or densities of all valid spots on the gels. Only spots with a 1.5-fold increase or decrease in normalized spot density in those samples and a statistically significant difference based on a Student's *t*-test at 95% confidence (i.e., *p<*0.05) were considered for MS/MS analysis.

### In-gel trypsin digestion

In-gel trypsin digestion of selected gel spots was performed as previously described [23]. Briefly, protein spots identified as significantly altered were excised from 2D-gels with a clean, sterilized blade and transferred to Eppendorf microcentrifuge tubes. Gel plugs were then washed with 0.1 M ammonium bicarbonate NH_4_HCO_3_) at RT for 15 min, followed by incubation with 100% acetonitrile at RT for 15 min. After solvent removal, gel plugs were dried in their respective tubes under a flow hood at RT. Plugs were incubated for 45 min in 20 μl of 20 mM DTT in 0.1 M NH_4_HCO_3_ at 56°C. The DTT/NH_4_HCO_3_ solution was then removed and replaced with 20 μl of 55 mM iodoacetate (IA) solution in 0.1 M NH_4_HCO_3_ and incubated with gentle agitation at room temperature in the dark for 30 min. Excess IA solution was removed and plugs incubated for 15 min with 200 μl of 50 mM NH_4_HCO_3_ at RT. A volume of 200 μl of 100% acetonitrile was added to this solution and incubated for 15 min at room temperature. Solvent was removed and gel plugs were allowed to dry for 30 min at RT under a flow hood. Plugs were rehydrated with 20 ng/μl of modified trypsin (Promega, Madison, WI, USA) in 50 mM NH_4_HCO_3_ in a shaking incubator overnight at 37°C. Enough trypsin solution was added in order to completely submerge the gel plugs.

### Mass spectrometry (MS)

Salts and contaminants were removed from tryptic peptide solutions using C18 ZipTips (Sigma-Aldrich, St. Louis, MO, USA), reconstituted to a volume of ∼15 μl in a 50∶50 water: acetonitrile solution containing 0.1% formic acid. Tryptic peptides were analyzed with an automated Nanomate electrospray ionization (ESI) [Advion Biosciences, Ithaca, NY, USA] Orbitrap XL MS (Thermo-Scientific, Waltham, MA, USA) platform. The Orbitrap MS was operated in a data-dependent mode whereby the eight most intense parent ions measured in the Fourier Transform (FT) at 60,000 resolution were selected for ion trap fragmentation with the following conditions: injection time 50 ms, 35% collision energy, MS/MS spectra were measured in the FT at 7500 resolution, and dynamic exclusion was set for 120 s. Each sample was acquired for a total of ∼2.5 min. MS/MS spectra were searched against the International Protein Index (IPI) database using SEQUEST with the following parameters: two trypsin miscleavages, fixed carbamidomethyl modification, variable methionine oxidation, parent tolerance 10 ppm, and fragment tolerance of 25 mmu or 0.01 Da. Results were filtered with the following criteria: Xcorr1.5, 2.0, 2.5, 3.0 for 1, 2, 3, and 4 charge states, respectively, Delta CN0.1, and *P*-value (protein and peptide) 0.01. IPI accession numbers were cross-correlated with Swiss Prot accession numbers for final protein identification.

### Statistical analysis

All statistical analyses were performed using a Mann-Whitney U statistical test and a two-tailed Student's *t*-test. p<0,05 was considered significant for differential fold-change values. Only proteins with significant p-values from *both* tests were considered further for MS identification. Protein and peptide identifications obtained with the SEQUEST search algorithm with p<0.01 were considered statistically significant. To further validate SEQUEST identification, the location of protein spots (i.e., molecular weight [MW] and isoelectric point [pI]) on 2D-gels was manually checked based on expected MW and pI values from SwissProt database information.

## Results

### Proteomics

Proteomics analysis using 2-DE and Sypro Ruby staining was performed on proteins isolated from brain mitochondria of WT and p53^(−/−)^ mice to determine proteins differently expressed. Fig. 1 shows 2D-gel images related to these analyses, with expanded images of protein spots significantly different (p<0.05) between WT and p53^(−/−)^. Twelve proteins were identified as differently expressed between WT and p53^(−/−)^ mice, and interestingly all twelve of these proteins were significantly over-expressed in p53^(−/−)^ samples. Surprisingly, we did not find any mitochondrial proteins down-regulated in p53^(−/−)^ mice relative to WT. The protein spots of interest were excised from the gels, and following digestion with the trypsin peptide were subjected to MS/MS analyses. Proteins identified are listed in Table 1 with the number of peptide sequences, the score, the coverage, MW, pI, fold-change levels, and p-value. All protein identifications were consistent with comparison of protein positions on the gel with MW and pI from databases.

The identified proteins were: guanine nucleotide-binding protein G (o) subunit alpha (212-fold ↑p53KO, *P<0.0019), ATP synthase subunit beta (125-fold ↑p53KO, *P<0.0035), heat shock cognate 71 (212-fold ↑p53KO, *P<0.002), aldehyde dehydrogenase family 5, subfamily A1 (131-fold ↑p53KO, *P<0.0009), glutamate dehydrogenase 1 (131-fold ↑p53KO, *P<0.0076), mitochondrial isoform of fumarate hydratase (325-fold ↑p53KO, *P<0.0019), acetyl-CoA acetyltransferase (166-fold ↑p53KO, *P<0.00079), isoform Mt-VDAC1 of voltage-dependent anion-selective channel protein 1 (201-fold ↑p53KO, *P<0.0027), aspartate aminotransferase (210-fold ↑p53KO, *P<0.0037), Mn superoxide dismutase (133-fold ↑p53KO, *P<0.0026), cytochrome b-c1 complex Rieske subunit (252-fold ↑p53KO, *P<0.0030), and thioredoxin-dependent peroxide reductase (253-fold ↑p53KO, *P<0.0015).

## Discussion

Several studies have described p53, an important tumor suppressor protein, as the guardian of the genome [1], [2] for its critical role in regulating the transcription of numerous genes responsible for cells cycle arrest, senescence, or apoptosis in response to various stress signals [4]. Therefore, p53 is crucial in maintaining genetic stability [1]. What determines cell fate is unclear but different factors including the cell type, the particular insult, and the severity of damage are involved in this decision [24].

Undoubtedly p53 promotes longevity by decreasing the risk of cancer through activation of apoptosis or cellular senescence, but several reports suggest that an increase of its activity may have detrimental effects leading to selected aspects of the aging phenotype [7], [25] and neurodegenerative disease. Thus, there is a balance between cell death and survival that under normal conditions optimizes tumor suppression without accelerating aging.

Previous research from our laboratory found p53 over-expressed and oxidatively modified by oxidative and nitrosative stress in brain from subjects with mild cognitive impairment (MCI) and AD brain, compared to control samples [26], [27]. Conformational alterations of p53 in MCI and AD are known [19]. These observations are consistent with the role played by p53 in neuronal death detected in neurodegenerative conditions, and with an important link of p53 with oxidative stress. ROS and p53 appear to be interconnected at multiple levels in their signaling pathways. First, ROS are potent activators of p53, acting in different ways such as damaged DNA, and even by regulating the redox status of cysteines present in the DNA-binding domain of p53, affecting its DNA-binding activity [26], [28], [29]. Moreover, once activated p53 generates downstream ROS which mediate apoptosis [12], [30]. Therefore p53 appears to regulate cellular redox status [11].

Since oxidative stress has been considered a crucial factor that contributes to neurodegenerative processes like AD [31]–[33], p53 could be a therapeutic target to reduce the levels of ROS, and in this way prevent or attenuate neuronal death in neurodegenerative disorders such as MCI and AD.

In a previous study, we demonstrated for the first time that the lack of p53 significantly decreases basal levels of oxidative and nitrosative stress in mice brain, and that this loss of p53 could activate diverse protective pathways involved in maintaining cellular homeostasis in the brain of p53^(−/−)^ mice [20]. In the present study using proteomics, we gained insight into the role of p53 in the CNS, and tested the hypothesis that knock out of p53 affected the expression of several brain mitochondrial proteins involved in different pathways; thus, loss of p53 may present a target to restore neuronal impairment. Since our investigation was performed on isolated brain mitochondria from p53^(−/−)^ mice, our results conceivably could provide insights into progression of many mitochondrial-associated diseases. Hence, the identified proteins are involved in energy and mitochondrial alterations, signal transduction, antioxidant defense, and chaperone proteins, as shown in Table 2.

### Antioxidant defense

Interestingly, MnSOD was significantly increased in mitochondria isolated from the brain of p53^(−/−)^ mice compared to WT. This data was already shown in our prior study [20] and are consistent with the notion that MnSOD is transcriptionally repressed by p53 [34], [35] with consequent propagation of oxidative stress, since MnSOD provides critical antioxidant defense. Because the apoptotic programs require oxidative stress for their execution, an overexpression of MnSOD was shown to increase resistance to p53-dependent apoptosis [17], [34]. Drane et al. [34], and St. Clair and colleagues [18], further demonstrated that MnSOD has a mutual activity on p53 reducing its expression, and even negatively modulating its apoptotic function. Several studies indicate that overexpression of MnSOD protects neurons from oxidative damage thus exerting a defensive role during AD development [36]. St. Clair and co-workers [36], using APP-PS-1 neurons as a model of AD, found a reduction of MnSOD expression during neuronal maturation with high levels of oxidative stress. These researchers also indicated p53 as a possible factor for the suppression of MnSOD [36]. Therefore, an overexpression of MnSOD through the inhibition of p53 could be helpful to prevent or slow the progression of neurodegenerative processes such as AD.

Thioredoxin-dependent peroxide reductase, also called peroxiredoxin 3, is an antioxidant protein localized mainly in the matrix of mitochondria, and it regulates physiological levels of H_2_O_2_ [37]. The peroxiredoxin system requires a family of proteins called sestrins for its regeneration [38], and sestrin expression is regulated by p53 [39], [40]. Previous studies showed that p53 upregulates the expression of sestrins, including peroxiredoxin [14]. In contrast, in our study, we found an increase of Prdx3 levels in the mitochondrial of p53^(−/−)^ mice, and a plausible explanation of this result could be, as proposed in our previous work [20], that the lack of p53 could disturb cellular homeostasis causing the activation of protective pathways by cells to combat cellular damage. Since H_2_O_2_ plays a central role in induction of apoptosis [41], the reduction of mitochondrial levels of H_2_O_2_by overexpression of Prdx3 seems to be antiapoptotic [42], and therefore beneficial for preserving cell survival. In addition Prdx3 was previously found down-regulated in AD brain [43].

### Chaperone proteins

Heat shock cognate (HSC)-71, a member of the Hsp70 family of proteins [44], was found up-regulated in the mitochondrial fraction isolated from the brain of p53^(−/−)^ mice compared to WT. Previously, Agoff [45] established that Hsp70 is repressed by p53, corroborating our result.

The Hsp family acts as chaperones assuring proper folding and assembly of proteins, and protects cells against apoptosis [46]. This latter function is prominently carried out by Hsp70. It is conceivable that the Hsp family exerts a crucial role in neuronal death linked with neurodegenerative disorders. HSC-71 is the constitutive isoform of the Hsp 70, activated by cells in adverse conditions. This chaperone protein is involved in the degradation of damaged proteins shuttling them for proteolysis [47]. In AD, the expression of Hsps seems to have a protective function to prevent the formation of amyloid fibrils [48], and previously, HSC-71 was found down regulated [49], and oxidatively modified in AD brain [50]. Therefore the increase of HSC-71 expression levels, induced by the lack of p53, conceivably could play a protective role in AD progression.

### Energy dysfunction and mitochondrial alterations

Several findings suggest that p53 has a role in the regulation of pathways involved in glucose metabolism, supporting oxidative phosphorylation and the pentose phosphate shunt, and inhibiting glycolysis [11]. These activities of p53 prevent cancer development. In addition, mitochondria are a major site in which some constituents of these pathways play a role. Therefore, there is a connection between p53 and mitochondria [51], and a better understanding of this link conceivably could provide insight into the progression of mitochondria related disorders.

In our study VDAC was found up-regulated in mitochondria of p53^(−/−)^ mice compared to mitochondria from WT mice. VDAC is a component of the mitochondria permeability transition pore (MPT), which allows the exchange of metabolities like ATP in and out of mitochondria, and it is also involved in synaptic communication and in the early phases of apoptosis [52]. Previous studies revealed the anti-apoptotic function of VDAC through its ability to bind BAK, a pro-apoptotic protein [53]. Likewise, VDAC may restrain p53, reducing its levels [54]. Therefore, these prior results suggest that VDAC and p53 are interconnected, and that lack of p53 could increase the expression of VDAC, in according with our results. The upregulation of VDAC conceivably could improve synaptic transmission and cell survival as well as modulate apoptotic events.

In addition, in our study we found several energy-related proteins: ATP synthase subunit beta, mitochondrial isoform of fumarate hydratase, and cytochrome bc1 complex Rieske subunit, over-expressed in brain mitochondrial of p53^(−/−)^ mice. Since inhibition of p53 leads to dependence of cells on glycolysis and to considerable impairment of aerobic pathways [55], our data may reflect a stress response to compensate for this effect. Moreover the p53-dependent protein targets may be highly cellular type specific. Accordingly, our results also may reflect the high glycolytic metabolism in brain. The over-expression of these proteins, involved in energy metabolism, seems to confirm the hypothesis of this work, in which diminution of p53 may represent a target to restore mitochondrial dysfunction, since these proteins were found altered in models of aging and neurodegenerative diseases [56]–[58].

p53 plays an additional role in the regulation of glutamate metabolism activating the expression of glutaminase 2 which provides glutamate to promote the tricarboxylic acid (TCA) cycle and oxidative phosphorylation [59]. Glutamate may be oxidatively deaminated by glutamate dehydrogenase to form α-ketoglutarate, which can then enter the Krebs cycle and be oxidized to CO_2_ and H_2_O, or α-ketoglutarate can be transaminated by aspartate aminotransferase to form the neurotransmitter glutamate. Both of glutamate dehydrogenase and aspartate aminotransferase were shown up-regulated in mitochondrial brain of p53 knockout mice. These data are consistent with our previous results showing the enhancement of aerobic pathways in p53-deficient mice [20], and their contrast with the current literature [60] can be explained by the notion that p53-dependent effects cannot be reproduced in a particular cell system. Previously, glutamate dehydrogenase and aspartate aminotransferase have been shown to be oxidatively modified, and expressed differently in animal models of neurodegeneration [61]–[63]. Therefore, even these results strongly support the concept that inhibition of p53 may attenuate neurodegenerative disorders.

Another notable mitochondrial protein found to be basally up-regulated in brain mitochondria of p53^(−/−)^ mice was aldehyde dehydrogenase family 5, subfamily A1, a member of the aldehyde dehydrogenase (ALDH) family known to participate in oxidizing a plethora of endogenous and exogenous aldehydes [64]. Previous studies showed a prominent role of ALDH family, including ALDH1, ALDH2, ALDH3A, and ALDH5A, in the oxidation of 4-hydroxy-*trans*-2-nonenal (HNE) to 4-hydroxy-*trans*-2-nonenoate (HNEAcid) [65], [66], a major pathway of HNE detoxification. In particular, in rat and in human brain, HNEAcid formation occurs in the mitochondria by ALDH5A [65]. The detoxification of these aldehydes is important for neurodegenerative disorders such as AD, since high levels of unsaturated lipid content increase brain vulnerability to oxidative damage [67]. Therefore up-regulation of ALDH5 could be protective against cell damage. Previous research identified ALDH4 as a p53-inducible gene [68]. The current study is the first to show that the lack of p53 increases ALDH5 expression levels, adding an additional p53-target gene.

An increasing body of evidence places p53 as a member of an intriguing network that includes tumor suppression and aging [69]. The tumor suppressor activity of p53 protects against malignant transformation but also enhances the aging process [69]. As the role of p53 in aging is still unclear, we designed this study gain insights into the role of p53 in the brain and its involvement in neuronal cell death.

In conclusion, we identified brain mitochondrial proteins in p53 null mice that display crucial p53-dependent cellular functions in the central nervous system. Therefore, our results reinforce the concept that the lack of p53 could disturb cell homeostasis causing cells to stimulate defensive pathways. We also elaborated on the link between p53 and mitochondria as we used for the study mitochondria from p53 knock out mice. Since mitochondrial dysfunction is a key feature of neurodegenerative diseases such as AD, p53 conceivably could be a novel therapeutic target for the treatment of these disorders (Fig. 2).
